# Identification of *4CL* Genes in Desert Poplars and Their Changes in Expression in Response to Salt Stress

**DOI:** 10.3390/genes6030901

**Published:** 2015-09-18

**Authors:** Cai-Hua Zhang, Tao Ma, Wen-Chun Luo, Jian-Mei Xu, Jian-Quan Liu, Dong-Shi Wan

**Affiliations:** State Key Laboratory of Grassland Agro-Ecosystem, School of Life Sciences, Lanzhou University, No.222, Tianshui Southern Road, Lanzhou 730000, China; E-Mails: chzhang13@lzu.edu.cn (C.-H.Z.); matao.yz@gmail.com (T.M.); wchluo2011@gmail.com (W.-C.L.); xujianmei131412@126.com (J.-M.X.); liujq@lzu.edu.cn (J.-Q.L.)

**Keywords:** *4CL* gene family, desert poplars, adaptive evolution, expression divergence, salt stress

## Abstract

4-Coumarate:CoA ligase (*4CL*) genes are critical for the biosynthesis of plant phenylpropanoids. Here we identified 20 *4CL* genes in the genomes of two desert poplars (*Populus euphratica* and *P*. *pruinosa*) and salt-sensitive congener (*P*. *trichocarpa*), but 12 in *Salix suchowensis* (*Salix willow*). Phylogenetic analyses clustered all Salicaceae *4CL* genes into two clades, and one of them (corresponding to the *4CL*-like clade from *Arabidopsis*) showed signals of adaptive evolution, with more genes retained in *Populus* than *Salix* and *Arabidopsis*. We also found that *4CL12* (in *4CL*-like clade) showed positive selection along the two desert poplar lineages. Transcriptional profiling analyses indicated that the expression of *4CL2*, *4CL11*, and *4CL12* changed significantly in one or both desert poplars in response to salt stress compared to that of in *P*. *trichocarpa*. Our results suggest that the evolution of the *4CL* genes may have contributed to the development of salt tolerance in the two desert poplars.

## 1. Introduction

The enzyme 4-coumarate:CoA ligase (*4CL*; EC 6.2.1.12) plays an important role in phenylpropanoid metabolism by catalyzing the formation of CoA ester [1,2]. *4CL* regulates a specific branched pathway that contributes to flavonoid and lignin synthesis [3]. Both of these products control various physiological functions in plants and improve plant adaptations to environmental stress [4]. *4CL* proteins belong to the AMP-binding protein family, containing one defining structural characteristic: two conserved domains within the AMP-binding domain [5]. One domain (Box I) consists of a serine/threonine/glycine (STG)-rich region followed by a proline/lysine/glycine (PKG) triplet [6], which is an important criterion in establishing an adenylate-forming superfamily [7]. Another domain includes a GEICIRG motif (Box II), whose central cysteine residue can be involved directly in catalysis [8].

Since the first *4CL* gene was cloned and identified in 1981 [9], a series of *4CL* and *4CL*-like genes have been found and investigated in many plant species, such as rice [10], soybean [11], loblolly pine [12], *Arabidopsis* [13,14], tobacco [15,16], aspen [2,17], and hybrid poplar [18]. 4CL is encoded by a multi-gene family in higher plants, and the number of gene members varies according to plant species. Based on phylogenetic analyses, *4CL* genes are divided into two classes, Class I and Class II [14,19]. The structure of Class I proteins is conserved across all plants, while that of Class II varies even within the same species [14]. In the *Arabidopsis* genome, four *4CL* genes and nine *4CL*-like genes have been detected; three *4CL* genes (*4CL1*, *4CL2* and *4CL4*) belong to Class I and one (*4CL3*) belongs to Class II, while the remaining nine genes are classified as *4CL*-likes [20], which were predicted to encode proteins nearly 50% identical over their full length to *4CL*s and contain several same conserved motifs as *4CLs* [19]. All of them are here treated as members of the same gene family. It has been suggested that the *4CL* genes in Class II are closely associated with flavonoid biosynthesis, while those in Class I are involved in the biosynthesis of lignin and other phenylpropanoids [14,19]. Even though their precise roles remain unknown, the *4CL*-like genes may be associated with other functions [19,20].

There is evidence that salt stress causes increased lignification of the cell wall in plants [21]. It has, therefore, been hypothesized that the functions of the *4CL* genes are closely linked to the environmental stresses that plants encounter [8,22,23]. Two poplar species (*P*. *euphratica* and *P*. *pruinosa*) that occur in desert regions have adapted to the high saline underground water and drought environment; both of these species play key roles in maintaining local arid ecosystems [24,25,26,27]. The wood of the two species is much harder than in other poplars [28], and they may have accumulated more lignin in the xylem for both structural support and water transport in response to their extremely arid environments. However, there has been limited research on this phenomenon. In this study, we carried out evolutionary analyses of the *4CL* genes of these two desert poplars and one salt-sensitive poplar (*P*. *trichocarpa*). We also looked for any change in expression of the *4CL* genes in this family in response to salt stress using calluses developed from three species. Our results provide important insights into the evolutionary roles of this gene family during the environmental adaptation of desert poplars.

## 2. Materials and Methods

### 2.1. Gene Sequence Collection and Identification of 4CL Genes

*4CL* genes were identified in the protein databases of *P*. *euphratica* [29], *P*. *trichocarpa* [30] (JGI v9.0) [31], *P*. *pruinosa* [32] and *Salix suchowensis* [33] using the reciprocal BLAST technique with protein sequences from the 13 *4CL* genes of *Arabidopsis thaliana*, retrieved from Cell Wall Genomics [34]. Poplar gene models homologous with the 13 *Arabidopsis* genes were sought based on these protein sequences using the program BLASTP with an e-value cut-off of 1-E30. The protein sequences resulting from positive poplar gene model hits then completed the BLAST search against all the proteins in the *Arabidopsis* genome from the TAIR 9.0 website [35]. Proteins that had one of the 13 *Arabidopsis* genes as a top-three hit were identified as candidate *4CL* proteins. We then applied the HMMER program [36] to identify *4CL* sequences in the poplar genomes using the AMP-binding domain sequence as a query, which was acquired using the Hidden Markov Models (HMM) profile [36] for the AMP-binding domain (PF00501.21) from the Protein Families database (PFAM) database [37]. These sequences were further verified using a PFAM batch search with default settings. Sequences that were confirmed by both methods were used for further analyses. The *4CL* genes used in this study were re-named based on the names of *Arabidopsis* genes in previous studies [20] and the phylogenetic analyses in this study. Information regarding all *4CL* genes from the five species used is given in Table S1.

### 2.2. Phylogenetic Analysis

Full-length amino acid sequences from *A*. *thaliana*, *P*. *euphratica*, *P*. *trichocarpa*, *P*. *pruinosa*, and *S*. *suchowensis* were aligned using CLUSTALW2 [38]. An un-rooted neighbor-joining (NJ) [39] tree was constructed using MUSCLE [40] and the Molecular Evolutionary Genetics Analysis version 6.0. (MEGA 6) package [41]. The tree nodes were evaluated by bootstrap analysis with 100 replicates. Branches with bootstrap values less than 50% were collapsed. In addition, gene structures were obtained by comparing coding regions and genomic sequences, and displayed using Scalable Vector Graphics (SVG) [42]. The putative *4CL* protein sequences used for the phylogenetic analysis were detected using Multiple Expectation Maximization for Motif Elicitation (MEME v4.10.1) [43] and applied to analyze possible conserved motifs; default parameters were used, except for the maximum number of identified motifs, which was defined as 20. The motifs were numbered according to their order displayed in MEME. The detected motifs were annotated using Simple Modular Architecture Research Tool (SMART) protein analyzing software [44,45].

### 2.3. Molecular Evolution Analysis

To evaluate variation in selective pressure in the two major clades that we identified, branch-specific models of CODEML in Phylogenetic Analysis by Maximum Likelihood (PAML v4.6) [46] were used to estimate the ratio of non-synonymous *vs*. synonymous substitutions (ω) under two *a priori* assumptions: a one-ratio model, in which one ω value was assumed for the entire tree, and a two-ratio model, in which ω values were allowed to vary between the two major clades.

**Figure 1:**
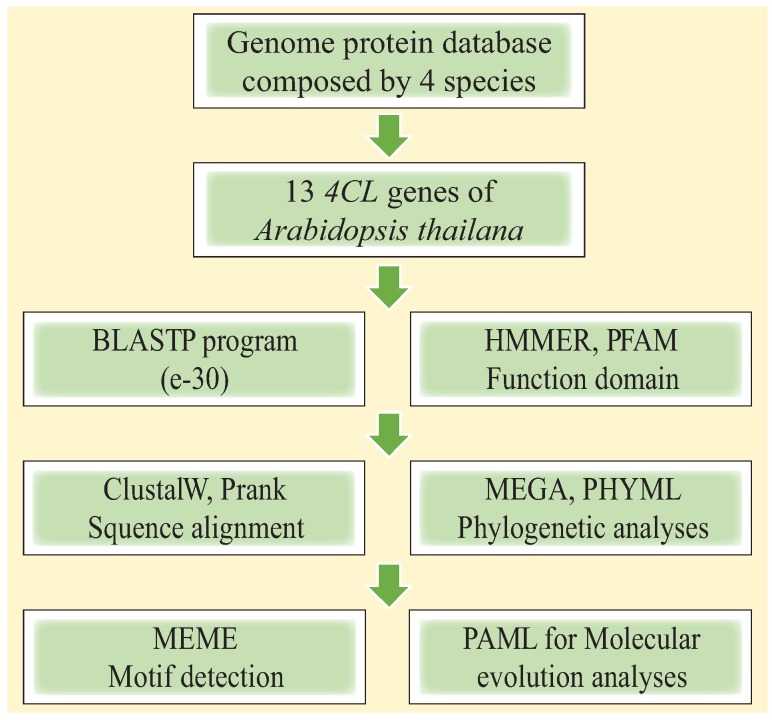
Schema of the experiment.

Identification of orthologous proteins between the five species was performed using the INPARANOID [47] and MULTIPARANOID programs [48]. Phylogenetic relationships among the orthologous groups were reconstructed using the MEGA 6 package. To evaluate variation in selective pressure across each orthologous group, the branch models of CODEML and PAML were used to estimate ω under different assumptions, by selecting *P*. *euphratica* or *P*. *pruinosa* as the foreground. For some orthologous groups, the branch-model tests indicated that selective pressures differed significantly between the two clades. Thus, we used branch-site models to test whether positive selection had occurred at some amino acid sites in the clade comprising *P*. *euphratica* and *P*. *pruinosa*. The Bayes Empirical Bayes method [49] was then applied to identify positively selected candidate sites. To examine which of the models fit the data best, Likelihood Ratio Tests (LRTs) were performed by comparing the difference in log likelihood values between pairs of models using a χ2 distribution, with the degrees of freedom equal to the difference in numbers of parameters between the models [50]. The flowchart for the *4CL* gene family analysis is summarized in Figure 1.

### 2.4. Expression of 4CL Genes in Salt Stress and Control Conditions

The expression analysis of poplar *4CL* genes was performed based on the RNA-Seq data from *P*. *trichocarpa* [51], *P*. *euphratica* [29], and *P*. *pruinosa* [52]. The calluses were firstly induced from shoots for each species using the methods described previously [53,54]. Then the total RNAs were isolated from the control and salt-stressed calluses (200 mM NaCl for 6, 12, 24, and 48 h) using a CTAB procedure [55]. The quality and integrity of the RNA samples were examined using the Agilent 2100 Bioanalyzer and their RIN (RNA Integrity Number) values ranged from 8.6 to 10.0, with no sign of degradation. Three biological replicates were conducted and equal quantities of total RNA were pooled for cDNA libraries construction. Poly(A) mRNA was isolated using beads with oligo (dT) before the mRNA was fragmented and cDNA synthesis performed using random hexamer-primers and reverse transcriptase (Invitrogen, Carlsbad, CA, USA). After end repair, adapter ligation and PCR amplification, the libraries were sequenced using an Illumina Genome Analyzer platform.

The cleaned reads from each of the three poplar species were mapped onto its own reference sequences using Bowtie2 (version 2.1.0, University of Maryland, College Park, MD, USA) [56] software with default parameters. The sensitivity of RNA-Seq will be a function of both molar concentration and transcript length. By normalizing for RNA length and for the total read number in the measurement, the RPKM measure of read density reflects the molar concentration of a transcript in the starting sample [57]. Therefore, gene expression levels were measured as the number of reads per kilobase per million mapped reads (RPKM) on exon regions within a given gene [57] and to reduce effects of background transcription, we selected only genes that had RPKM ≥1 in sample from two or more time points for further analysis. To identify differentially-expressed genes (DEGs) in control callus and salt-stressed callus from *P*. *trichocarpa*, *P*. *euphratica* and *P*. *pruinosa*, the edgeR [58] was used to estimate the means and variances of raw read counts under a negative binomial distribution and used exact tests to identify differentially-expressed transcripts. After the *p*-value for each expressed genes were obtained by edgeR, we used the false discovery rate (FDR) to justify the *p*-value by the function *p.adjust* in R. If log2 (FPKMsalt/FPKMcontrol) > 1 or < −1, and the adjusted *p*-value (FDR) was <0.05, the genes were identified differentially expressed genes (DEGs) [59].

## 3. Results and Discussion

### 3.1. Identification of the 4CL Genes from Four Species of Salicaceae

The initial step in identifying the gene family members was to find candidate genes with a similarity search [60]. We used the BLASTP program to search a database of annotated protein sequences from the three poplar species and one willow species, resulting in 168, 528 protein sequences in total. We identified 20, 20, 20 and 12 *4CL* genes from *P*. *trichocarpa*, *P*. *euphratica*, *P*. *pruinosa*, and *S*. *suchowensis* genomes, respectively (Table 1 and Table S1). All three poplars had the same number of *4CL* genes, which was more than that of the single willow species. Both *Salix* and *Populus* species have experienced a common genome duplication. However, the increased number of genes within one genus compared with another in the same family could be the result of two possibilities: *Populus* may have retained more gene copies than *Salix* following genome duplication, or the copies may be derived from expansions through *Populus*-specific segmental and tandem duplications [30].

### 3.2. Gene Structure and Motif Identification of 4CL Genes in Poplars

**Figure 2:**
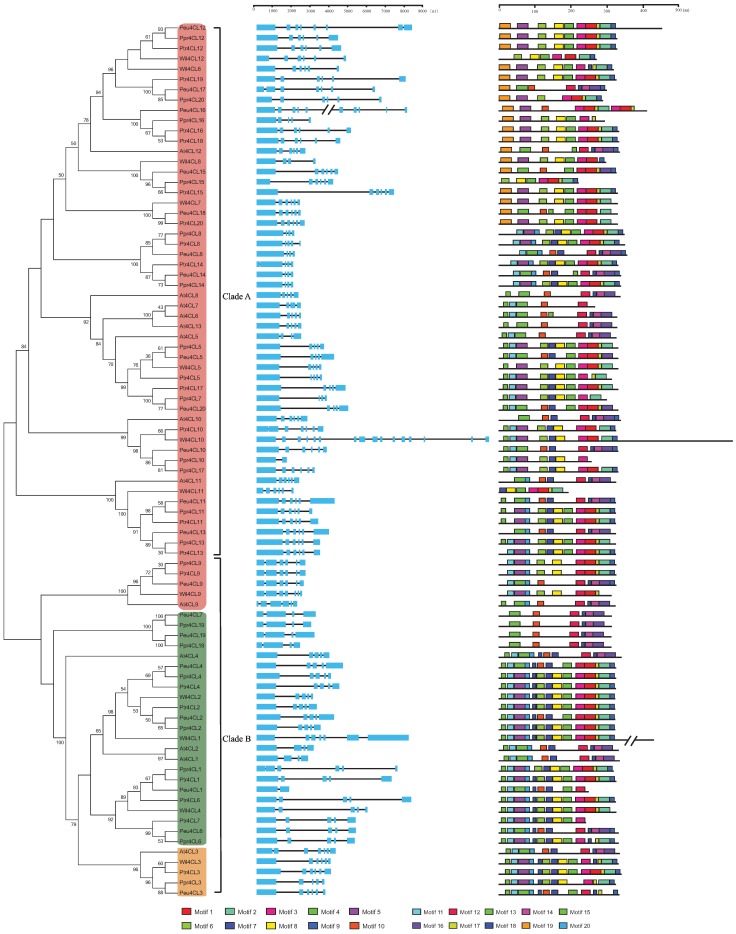
Phylogenetic relationships, gene structure and motif structure of *4CL* genes in the five considered species. An un-rooted NJ tree was constructed from the alignment of full-length amino acid sequences of *Arabidopsis thaliana* (At), *P*. *trichocarpa* (Ptr), *P*. *pruinosa* (Ppr), *P*. *euphratica* (Peu), and *Salix suchowensis* (Wil) using the MEGA 6 package. Branches with less than 50% bootstrap values were collapsed. The tree divided into two clades: designated Clades A and B. The different color backgrounds represent three kinds of *4CL*s: *4CL*-like (red), Class I (green), and Class II (orange). Lengths of exons and introns of each *4CL* gene are displayed proportionally. The blue solid boxes represent exons; black lines represent introns. Motifs represented with boxes were predicted using MEME. The numbers and colors represent motifs 1–20. Box size indicates the length of the motif.

To understand better the diversity of *4CL* genes in the Salicaceae, intron/exon arrangements and conserved motifs were compared using their phylogenetic relationships (Figure 2). As shown in Figure 2, closely related genes were generally more similar, structurally, differing only in intron and exon lengths. Some close gene pairs did have distinct intron/exon arrangements. For example, *Peu4CL16* had 10 exons, whereas its close orthologs *Ptr4CL16* and *Ppr4CL16* had only six and five, respectively, even though their phylogenetic relationship was supported by a high bootstrap value. However, overall, our results show that the *4CL* genes in poplars have relatively conservative exon/intron structures.

**Table 1:** Comparison of *4CL* gene family sizes in the five considered species.

Organisms	*4CL* groups			Total *4CLs*
	Class I	Class II	Class-*4CL* like	
*Arabidopsis thaliana*	3	1	9	13
*Populus trichocarpa*	5	1	14	20
*Populus pruinosa*	4	1	15	20
*Populus euphratica*	4	1	15	20
*Salix suchowensis*	3	1	8	12

We then used the online program MEME v4.10.1 to analyze conserved motifs in *4CL* proteins. A total of 20 conserved motifs were identified (Figure 2 and Table S2). Motif 6 and motif 10 were represented by the typical AMP-binging domain, which is rich in Gly, Ser, and Thr. The *4CLs* belonging to the superfamily of adenylate-forming enzymes contained motif 6 or motif 10. Motif 3 and motif 12 were the second most conserved signature motif, with GEICIRG. Their central cysteine residue is thought to be directly involved in catalysis [5]. In addition to the conserved domains, several other conserved motifs were common in all *4CL* genes, such as motifs 2, 4, and 13, indicating their importance. Then we subjected the motifs 2, 4, and 13 to SMART annotation. Motif 1 and Motif 4 were represented by the AMP-binding domain for AMP-dependent synthetase or ligase and Motif 13 was represented by the AMP-binding enzyme C-terminal domain.

### 3.3. Phylogenetic Analysis of 4CL Genes

In order to determine the evolutionary relationships between the poplar *4CL* proteins and *4CL* proteins known from other species, we performed multiple sequence alignment and generated a NJ phylogenetic tree for *4CL* proteins from *Arabidopsis*, *P*. *euphratica*, *P*. *pruinosa*, *P*. *trichocarpa*, and *S*. *suchowensis* (Figure 2). For this study, we generated a new version of the phylogenetic reconstruction that incorporated the *4CL* and *4CL*-like amino acid sequence data from three poplars, one willow, and *Arabidopsis*. In total, all 72 confirmed *4CL* genes from Salicaceae and 13 from *Arabidopsis* clustered into two clades, designated Clades A and B. Clade A included most of the *4CL*-like genes identified from *Arabidopsis* (*At4CL5-8*, *At4CL10-13*), while Clade B contained four *Arabidopsis 4CL* genes (*At4CL1*, *At4CL2*, *At4CL3*, and *At4CL4*), and one *4CL*-like gene (*At4CL9*). *At4CL3* was ascribed to *4CL* Class II [14] and our phylogenetic analyses identified only one orthologous copy for each poplar or willow with their common origin. However, for 4CL Class I [14], comprising At4CL1, At4CL2, and At4CL4, we recovered independent gene duplications for both Arabidopsis and Salicaceae. At4CL1 and At4CL2 clustered into one subclade and At4CL4 formed another separate subclade, while those from Salicaceae comprised two independent subclades. Numbers of 4CL Class I genes in the investigated species of Salicaceae ranged from three (*S*. *suchowensis*), through four (*P*. *euphratica* and *P*. *pruinosa*) to five (*P*. *trichocarpa*) (Table 1). For the *4CL*-like gene located in Clade B, *At4CL9*, there was only one homologous gene in each Salicaceae species. Within Clade A, eight *4CL*-like genes from *Arabidopsis* clustered, while varying numbers of copies were found in the Salicaceae species: 14 in *P*. *euphratica* and *P*. *pruinosa*, 13 in *P*. *trichocarpa*, and seven in *S*. *suchowensis*. Similarly, phylogenetic analyses indicated that most *4CL*-like genes from *Arabidopsis* and Salicaceae had common orthologous origins, but some were derived from an independent lineage-specific genome or gene duplications. These findings suggest that some *4CL* genes are evolutionarily dynamic while others have remained stable; the duplicated genes would have been lost after the common genome duplications. In total, across one salt-sensitive and two salt-tolerant poplars, we recovered six groups of 1:1:1 *4CL* orthologous genes. In addition, we found that independent duplication had occurred once for *P*. *trichocarpa* (*Ptr4CL16* and *Ptr4CL18*) and once for *P*. *pruinosa* (*Ppr4CL10* and *Ppr4CL17*), resulting in one more gene for each species in smaller subclades.

### 3.4. Molecular Evolution

The phylogenetic relationships among the *4CL* genes showed that a total of 85 full-length genes encoding putative 4CL proteins were grouped into two distinct clades (Figure 2). To determine whether there was a significant difference in selective pressure between Clades A and B (Figure 2), we performed a maximum likelihood codon model analysis using the PAML package. Two assumptions were tested: a one-ratio model that assumed the same ω (= *dN/dS*) ratio for both clades of *4CLs* and a two-ratio model in which the two *4CL* types were assigned different ω ratios. The log likelihood values under the one-ratio and two-ratio models were ln *L* = −8739.544694 and −8719.525874, respectively (Table 2). The likelihood ratio test indicated that the null (single ratio) model should be rejected and (thus) selective pressure has differed significantly between Clades A and B (*p* < 0.001). Under the two-ratio model, the ω values for Clades B and A were 0.21307 and 0.08243, respectively, indicating that Clade A (including the most *4CL*-like genes) has been under more relaxed selection constraints than Clade B (comprising four *4CL* genes, *4CL1-4*, and one homologous *4CL*-like gene, *4CL9*).

**Table 2:** Summary statistics of clades for detecting selection using branch models in PAML.

Branch model	ω	ln *L*	χ^2^	*p*
One-ratio	ω = 0.14106 for all branches	−8739.54		
Two-ratios	ω_0_ = 0.08243 for Clade I	−8719.53	40.04	< 0.001
	ω_1_ = 0.21307 for Clade II			
Two-ratios	ω_0_ = 0.08243 for Clade II			
	ω_1_ = 0.21307 for Clade I			

**Figure 3:**
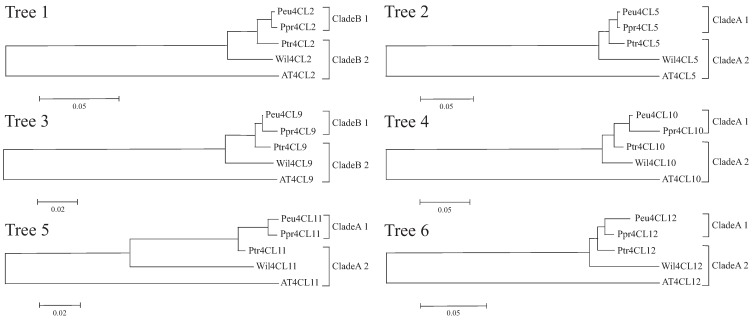
Phylogenetic trees of six orthologous *4CL* genes between four poplar species and *Arabidopsis* for molecular evolution analyses. The trees were reconstructed using NJ with 100 bootstrap replicates. Based on the orthologs belonging to Clade A or Clade B in Figure 2, A and B represent Clade A and Clade B, respectively, so the desert poplar clades (*P*. *pruinosa* and *P*. *euphratica*) were assigned to A1 or B1 and the other species were assigned to A2 or B2.

In order to detect evidence of adaptive evolution of the desert poplar clade comprising *P*. *pruinosa* and *P*. *euphratica* genes, we only used six groups of 1:1:1 orthologous genes across all five species for the analyses. We excluded groups with species-specific gene duplications or losses, which might affect the estimation of adaptive evolution. We carried out phylogenetic analyses for each group (Figure 3) and examined the significance of differences in apparent selection pressure between the desert poplar clade (*P*. *pruinosa* and *P*. *euphratica*) (A1 or B1) and the other species (A2 or B2). We used CODEML branch models in PAML to estimate ω (= *dN/dS*) values for the two clades (Table 3). An LRT showed that the two-ratio model provided a significantly better fit than the one-ratio model for trees five (*4CL11*) and six (*4CL12*) (*p* < 0.05), suggesting that there were significant differences in selective pressures between the two clades of *4CL* genes after the ancestral gene duplication. However, for the other four trees (trees 1, 2, 3, and 4), LRTs indicated that the one-ratio model (in which the two clades were assigned the same ratio) could not be rejected, suggesting that the selective pressure was similar between the two clades.

**Table 3:** Summary statistics of orthologs for detecting selection using branch models in PAML.

Tree	Branch model (tree)	ω	ln *L*	χ^2^	*p*
Tree 1					
	One-ratio	ω = 0.09995 for all branches	−4106.505177	3.473988	
	Two-ratios	ω_0_ = 0.12256 for Clade B1	−4104.768183		
		ω_1_ = 0.05817 for Clade B2			
Tree 2					
	One-ratio	ω = 0.14031 for all branches	−3879.08919	3.149266	
	Two-ratios	ω_0_ = 0.3196 for Clade A1	−3877.514557		
		ω_1_ = 0.1330 for Clade A2			
Tree 3					
	One-ratio	ω = 0.07578 for all branches	−4312.023369	0.768848	
	Two-ratios	ω_0_ = 0.10875 for Clade B1	−4311.638945		
		ω_1_ = 0.07299 for Clade B2			
Tree 4					
	One-ratio	ω = 0.19107 for all branches	−3400.606728	3.567392	
	Two-ratios	ω_0_ = 0.34271 for Clade A1	−3398.823032		
		ω_1_ = 0.17030 for Clade A2			
Tree 5					
	One-ratio	ω = 0.09507 for all branches	−2426.383206	7.862458	< 0.05
	Two-ratios	ω_0_ = 0.40447 for Clade A1	−2422.451977		
		ω_1_ = 0.08459 for Clade A2			
Tree 6					
	One-ratio	ω = 0.13484 for all branches	−3524.776614	5.583034	< 0.05
	Two-ratios	ω_0_ = 0.30109 for Clade A1	−3521.985097		
		ω_1_ = 0.12124 for Clade A2			

**Figure 4:**
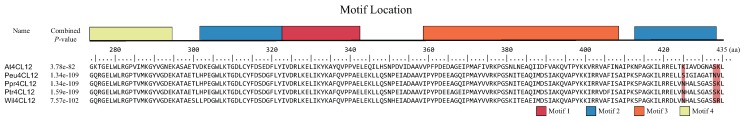
Positions of the positive selection sites. Sequence alignment of *4CL12* orthologs from 274–435 amino acid sites from five species included five motifs; the numbers and colors represent motifs 1–4. The three positive-selection sites predicted by the branch-site model test are colored red. Two sites (alignment positions 425 and 433) were located in motif 2. One site (alignment position 434) was located in none of the motifs.

For trees 5 and 6, in which selective pressures differed between the two clades, the mean ω values for Clades A1 and A2 were 0.40 and 0.08 or 0.30 and 0.12, respectively, indicating that more amino acid changes in the desert popular clade (A1) might have been preserved by positive selection. To test this hypothesis, we applied a branch-site test to identify target sites that were potentially under positive selection in Clade A1 of these two trees. We assigned Clades A1 and A2 as foreground and background branches, respectively, and obtained log likelihood values under the positive selection model A. We detected signals for tree 6 and the null model A0 of ln *L* = −3482.762192 and ln *L* = −3471.911359, respectively. The LRT indicated that the null model should be rejected (*p* < 0.05), corroborating the hypothesis that some amino acid sites in Clade A1 have been under positive selection for tree 6 (*4CL12*) (Table 4). Further analysis using a Bayes Empirical Bayes procedure identified three sites (alignment positions 425, 433 and 434) that have apparently been under positive selection with posterior probabilities >0.99. With one exception (alignment position 434), these amino acids were located in motif 2 (Figure 4).

**Figure 5:**
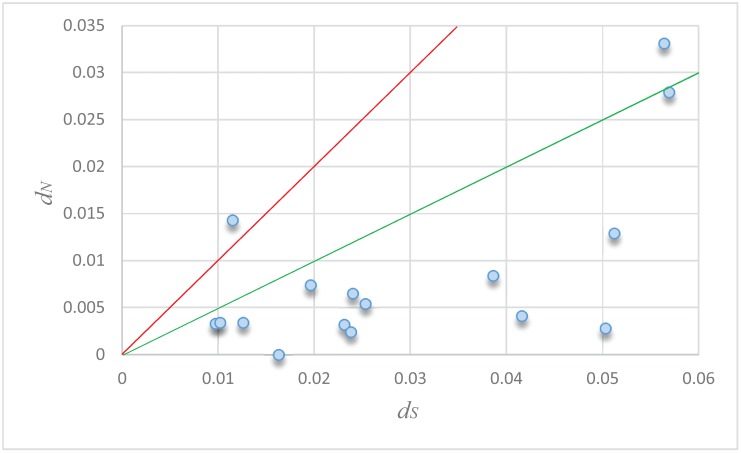
Distribution of *d_N_* and *d_S_* for 16 pairs of the putative *4CL* orthologs between *P*. *pruinosa* and *P*. *euphratica*. The orthologs with *d_N_*/*d_S_* > 1 fall above the red line while those with *d_N_*/*d_S_* = 0.5–1 fall between the green and red lines. The name and the value (*d_N_*/*d_S_*) of 16 pairs of genes were listed in the Table S3.

To infer the influence of selection on the pairs of orthologs from *P*. *euphratica* and *P*. *pruinosa*, the ratio of non-synonymous *vs*. synonymous substitutions (ω = *dN/dS*) was used because it is an indicator of the history of selection acting on a gene. Ratios significantly <1 are suggestive of purifying selection which hinders the spread of deleterious alleles, whereas ratios >1 suggest positive selection which promotes the spread of beneficial alleles in population [61]. A plot of *dN/dS* for the orthologous genes is shown in Figure 5 and the results suggest that most pairs have evolved mainly under the influence of purifying selection. Of these, one pair of orthologs (*Peu4CL15* and *Ppr4CL15*) between two desert poplars has a *dN/dS* > 1, indicating positive selection, and one pair has a *dN/dS* between 0.5 and 1, indicating weak purifying selection (Table S3).

**Table 4:** Summary statistics for detecting selection using branch-site models in PAML.

	Mode	Estimates of parameters	ln *L*	χ^2^	*p*	Positively selected sites
Tree6	Branch model	ω = 0.13484 for all branches	−3524.776614	5.583034	< 0.05	
	One-ratio	ω_0_ = 0.30109 for Clade A1	−3521.985097			
	Two-ratios	ω_1_ = 0.12124 for Clade A2				
	Branch-site model					
	Model A0	ω_0_ = 0.05407, p_0_ = 0.82301, ω_l_ = 0.05407, p_1_ = 0.17699	−3482.762192			
	(ω2 = 1)	ω_2a fore_ = 0.40000, ω_2a back_ = 0.05407, P_2a_ = 0.00000				
		ω_2b fore_ = 1.00000, ω_2b back_ = 0.40000, P_2b_ = 0.00000				
	Model A1	ω_0_ = 0.05457, p_0_ = 0.82708, ω_l_ = 1.00000, p_1_ = 0.15615	−3471.911359	21.701666	< 0.01	425 *, 433 **, 434 *
	(ω2 > 1)	ω_2a fore_ = 90.73377, ω_2a back_ = 0.05457, P_2a_ = 0.01411				
		ω_2b fore_ = 90.73377, ω_2b back_ = 1.00000, P_2b_ = 0.00266				

### 3.5. Changes in Expression of Orthologs Evolved under Salt Stress

**Figure 6:**
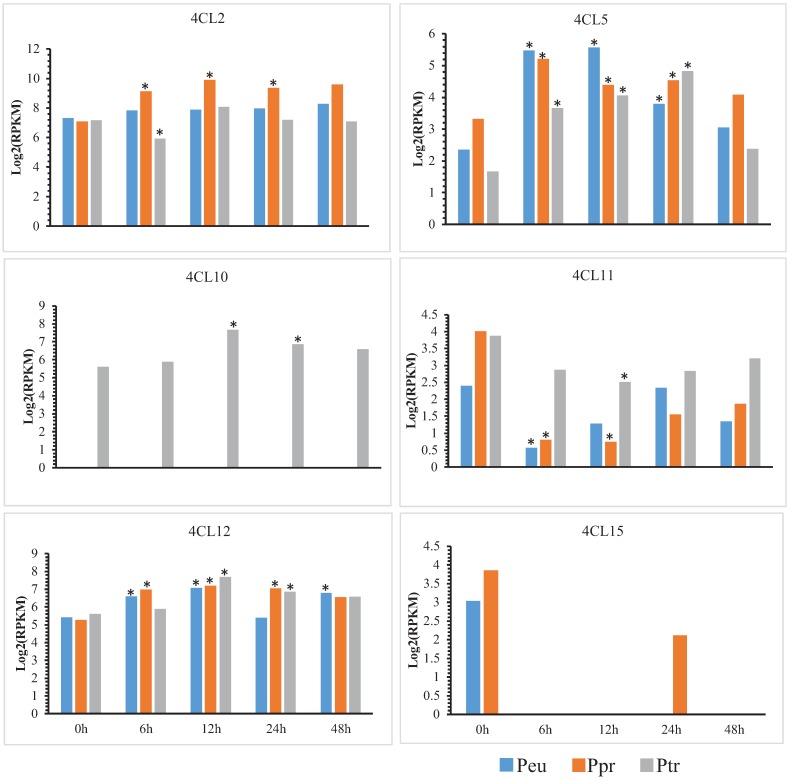
Expression analysis of the orthologous *4CL* genes under salt stress. Transcription analysis of the relative expression levels of six orthologous *4CL* genes specific to three species under salt stress except *4CL9*, which was not expressed in neither un-stressed nor stressed calluses, and the orthologous genes (*4CL15*) with ω (*dN/dS*) > 1 in *P*. *pruinosa* and *P*. *euphratica*. The y-axis shows the log2-transformed RPKM values of genes expressed in callus after different periods of salt stress, and the x-axis shows the time point under salt stress. The star (*) represents the expression of genes under salt stress that reached the threshold of log2 (RPKMsalt/RPKMcontrol) > 1 or < −1 and indicates a significance of *p* < 0.05.

We also examined possible differences in expression of the *4CL* genes between the salt-sensitive species *P*. *trichocarpa* and the two salt-tolerant species (*P*. *euphratica* and *P*. *pruinosa*) during responses to salt stress. Gene expression was measured in terms of RPKM values by mapping reads from the transcriptomes of control and salt-stressed calluses and it is very meaningful to do the similar experiments with different plant tissue or cuttings in the future. Thresholds of log2 (RPKM_salt_/RPKM_control_) > 1 and *p* < 0.05 were used to define significant differences in gene expression. Based on the thresholds and log_2_ ratio values, we found that expression levels of five *4CL* genes of the two salt-tolerant species (*P*. *euphratica* and *P*. *pruinosa*) significantly changed under salt stress, while the orthologous or homologous genes in the salt-sensitive *P*. *trichocarpa* showed similar but weaker changes. Expression levels of three *4CL* genes (*4CL2*, *4CL11* and *4CL12*) was significantly changed in one or both desert poplars in response to salt stress than in the salt-sensitive *P*. *trichocarpa* (Figure 6). However, expression of *4CL5* significantly decreased in response to salt stress in all three poplars. It should be noted that one of the three genes with an induced increased expression (*4CL12*) showed positive selection in the analyses described above. In contrast, a pair of rapidly evolved orthologs of the two desert poplars (*Peu4CL15* and *Ppr4CL15*, ω > 1) were only expressed under normal conditions (not under salt stress), suggesting that they also respond, negatively, to salt stress. In addition, we found that some *4CL* genes (e.g., *4CL9* and *4CL10*) were not expressed in either un-stressed or stressed calluses of any of the three poplar species. All these expression patterns suggest that some *4CL* genes may have played some roles in the evolution of salt-tolerance in *P*. *euphratica* and *P*. *pruinosa*. Furthermore, spatial and diachronic divergences in expression profiles of all *4CL* genes of the species have developed, suggesting possible functional divergences between them.

## 4. Conclusions

We have characterized the *4CL* gene family in poplars by a comprehensive analysis of gene structures, phylogenetic relationships, conserved motifs, molecular evolution and expression profiles. And we have identified 20, 20, 20 and 12 *4CL* genes from *P*. *trichocarpa*, *P*. *euphratica*, *P*. *pruinosa*, and *S*. *suchowensis* genomes, respectively. The *4CL* genes identified clustered into two clades, between which different selection pressures were detected. One gene (*4CL12* in the *4CL-like* clade) showed positive selection along the lineage comprising the two desert poplars. In addition, expression of three *4CL* genes (*4CL2*, *4CL11*, and *4CL12*) was induced substantially more strongly in one or both desert poplars in response to salt stress than in the salt-sensitive *P*. *trichocarpa*. Taken together, our findings suggest that the evolution of the *4CL* genes may have contributed to the development of salt tolerance in the two desert poplars.

## Supplementary Files

Supplementary File 1

## Data Deposit

The unpublished RNA-Seq data sets from *P*. *trichocarpa* of Illumina sequencing in are available at the NCBI Sequence Read Archive (SRA) database with the project accession number: PRJNA284202.
