# Supplementary material for: Identification of 4CL Genes in Desert Poplars and Their Changes in Expression in Response to Salt Stress
Source: Genes (Basel). 2015 Sep 18;6(3):901–17. doi: 10.3390/genes6030901 (PMC4584335; doi:10.3390/genes6030901)
Supplement: Supplementary File 1 [file genes-06-00901-s001.pdf]

Supplementary Information

Table S1. The list of *4CL* genes characterized in five species.

Species	Gene name	Gene ID	Length of protein (aa)	Number of intron
***Arabidopsis thailana*（At）**				
Class Ⅰ	*4CL1*	At1g51680	561	3
	4CL2	At3g21240	556	3
	4CL4	At3g21230	570	4
Class Ⅱ	*4CL3*	At1g65060	561	6
Class 4CL likes	*4CL5*	At1g20510	546	3
	4CL6	At1g20500	550	4
	4CL7	At1g20490	447	1
	4CL8	At1g20480	565	4
	4CL9	At1g62940	542	5
	4CL10	At4g19010	566	5
	4CL11	At4g05160	544	5
	4CL12	At5g63380	562	5
	4CL13	At5g38120	550	4
***Populus euphratica* (Peu)**				
Class Ⅰ	*4CL1*	CCG029167.1	417	1
	4CL2	CCG032591.1	540	4
	4CL4	CCG031380.1	544	4
	4CL6	CCG005472.1	556	4
Class Ⅱ	*4CL3*	CCG021432.1	560	5
Class 4CL likes	*4CL5*	CCG029325.1	554	4
	4CL7	CCG005977.1	524	3
	4CL8	CCG012691.1	596	3
	4CL9	CCG011911.1	545	5
	4CL10	CCG024728.1	554	5
	4CL11	CCG001141.1	543	5
	4CL12	CCG033354.1	757	7
	4CL13	CCG015470.1	543	5
	4CL14	CCG016610.1	567	3
	4CL15	CCG029088.1	546	5
	4CL16	CCG033353.1	687	9
	4CL17	CCG012530.1	501	6
	4CL18	CCG031826.1	552	5
	4CL19	CCG024486.1	522	3
	4CL20	CCG028829.1	554	4

Table S1. *Cont.*

Species	Gene name	Gene ID	Length of protein (aa)	Number of intron
Populus pruinosa **(Ppr)**				
Class Ⅰ	*4CL1*	PPR016303.1	534	5
	4CL2	PPR029403.1	540	4
	4CL4	PPR026307.1	544	4
	4CL6	PPR009676.1	556	4
Class Ⅱ	*4CL3*	PPR023820.1	543	4
Class 4CL likes	*4CL5*	PPR030299.1	554	4
	4CL7	PPR005839.1	501	3
	4CL8	PPR010421.1	583	3
	4CL9	PPR030819.1	545	5
	4CL10	PPR001615.1	431	1
	4CL11	PPR021586.1	543	5
	4CL12	PPR021750.3	550	5
	4CL13	PPR028238.1	1087	11
	4CL14	PPR029805.1	567	3
	4CL15	PPR018063.1	371	5
	4CL16	PPR021749.1	492	4
	4CL17	PPR009865.1	554	5
	4CL18	PPR013131.1	522	3
	4CL19	PPR015211.1	524	3
	4CL20	PPR012887.1	484	5
Populus trichocarpa **(Ptr)**				
Class Ⅰ	*4CL1*	Potri.006G169700.1	545	4
	4CL2	Potri.001G036900.1	540	4
	4CL4	Potri.003G188500.1	543	4
	4CL6	Potri.006G169600.1	543	4
	4CL7	Potri.018G094200.2	404	4
Class Ⅱ	*4CL3*	Potri.019G049500.2	570	5
Class 4CL likes	*4CL5*	Potri.002G012800.1	525	4
	4CL8	Potri.010G230200.1	585	4
	4CL9	Potri.001G055700.1	545	5
	4CL10	Potri.003G099700.3	544	6
	4CL11	Potri.017G112800.1	543	5
	4CL12	Potri.012G094800.1	550	5
	4CL13	Potri.004G102000.1	543	5
	4CL14	Potri.008G031500.1	555	4
	4CL15	Potri.017G033600.1	552	5
	4CL16	Potri.012G094900.1	558	5

Table S1. *Cont.*

Species	Gene name	Gene ID	Length of protein (aa)	Number of intron
	4CL17	Potri.005G248500.1	554	4
	4CL18	Potri.012G095000.1	558	5
	4CL19	Potri.015G092300.1	546	5
	4CL20	Potri.010G057000.1	552	5
Salix suchowensis (Wil)				
Class I	*4CL1*	willow_GLEAN_10006487	1490	6
	4CL2	willow_GLEAN_10023310	540	4
	4CL4	willow_GLEAN_10020544	545	4
Class II	*4CL3*	willow_GLEAN_10007365	556	5
Class *4CL* likes	*4CL5*	willow_GLEAN_10022611	554	4
	4CL6	willow_GLEAN_10016486	533	5
	4CL7	willow_GLEAN_10021672	552	5
	4CL8	willow_GLEAN_10003989	498	3
	4CL9	willow_GLEAN_10023426	523	6
	4CL10	willow_GLEAN_10025677	1084	15
	4CL11	willow_GLEAN_10018659	324	5
	4CL12	willow_GLEAN_10013755	454	5

Table S2. Details of motif sequences.

Motif	Length	Logo representation of the motif
1	50	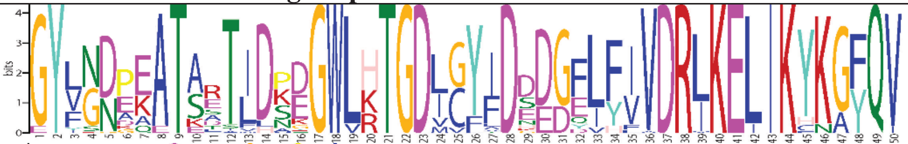
2	50	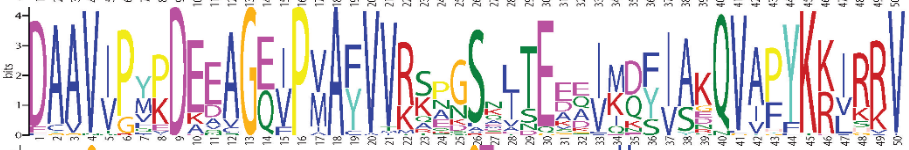
3	41	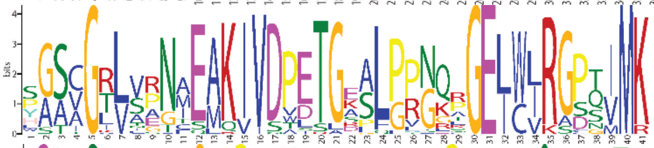
4	41	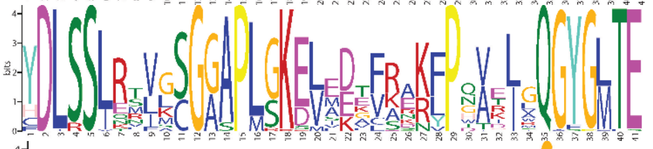
5	50	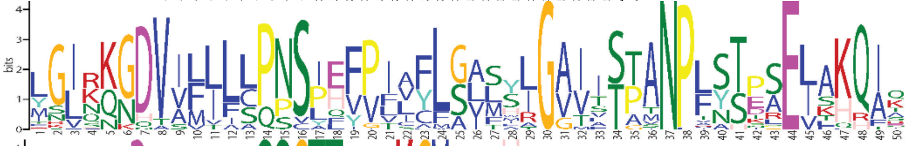
6	35	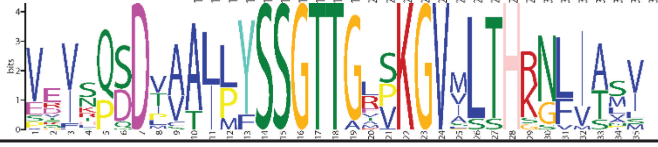

Table S2. *Cont.*

Motif	Length	Logo representation of the motif
7	29	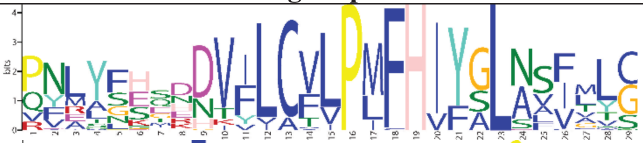
8	41	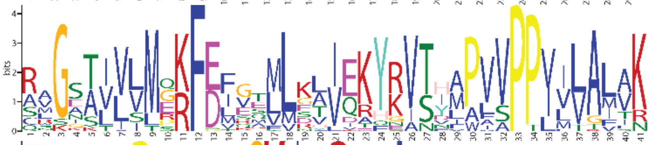
9	21	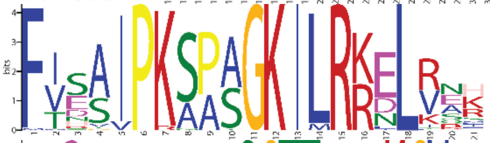
10	29	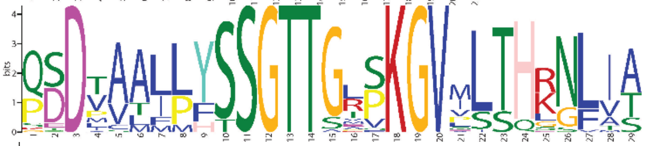
11	29	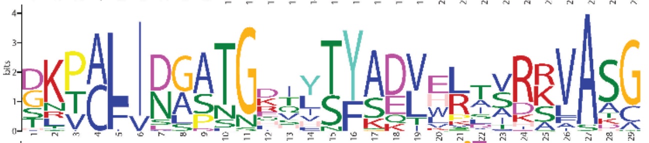
12	41	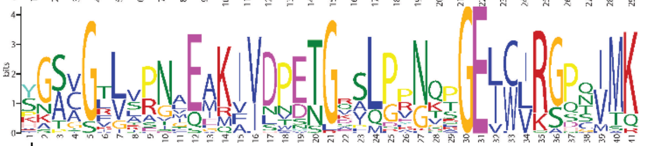
13	50	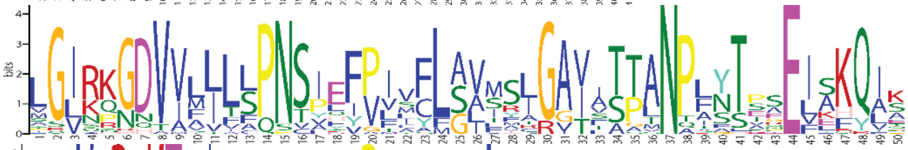
14	35	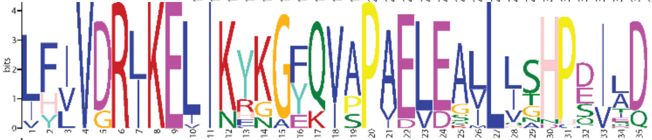
15	21	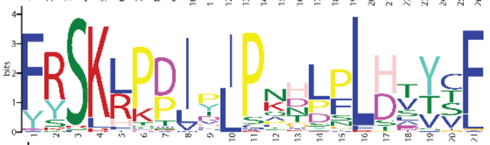
16	50	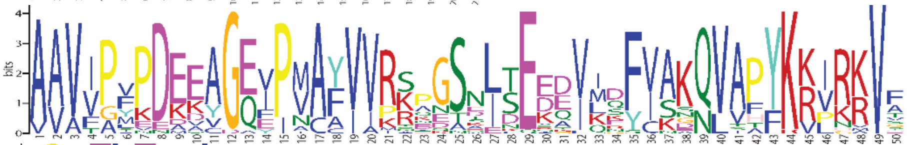
17	15	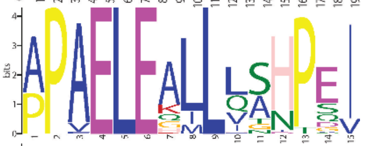
18	15	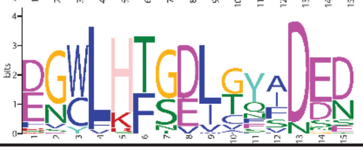

Table S2. *Cont.*

Motif	Length	Logo representation of the motif
19	50	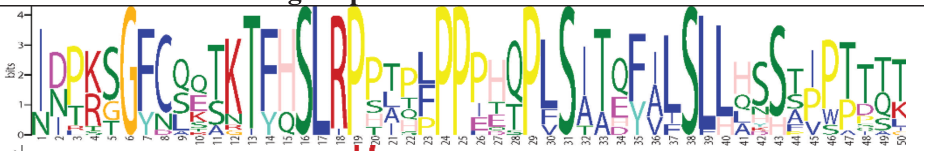
20	21	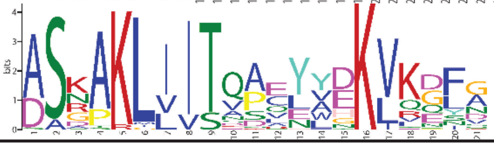

Table S3. The value of ω for 16 pairs of the putative orthologs.

Ppr	Peu	dN ± SE	dS ± SE	ω
PPR028238.1	CCG015470.1	0.0054 ± 0.0021	0.0253 ± 0.0079	0.2135
PPR021586.1	CCG001141.1	0.0033 ± 0.0017	0.0097 ± 0.0049	0.339
PPR030819.1	CCG011911.1	0.0032 ± 0.0016	0.0231 ± 0.0077	0.1402
PPR009676.1	CCG005472.1	0.0041 ± 0.0018	0.0416 ± 0.0099	0.0986
PPR016303.1	CCG029167.1	0.0279 ± 0.0056	0.0569 ± 0.0139	0.4899
PPR026307.1	CCG031380.1	0	0.0163 ± 0.0058	0
PPR029403.1	CCG032591.1	0.0024 ± 0.0014	0.0238 ± 0.0080	0.1024
PPR023820.1	CCG021432.1	0.0034 ± 0.0017	0.0126 ± 0.0056	0.2719
PPR010421.1	CCG012691.1	0.0084 ± 0.0025	0.0386 ± 0.0097	0.2168
PPR029805.1	CCG016610.1	0.0065 ± 0.0023	0.0240 ± 0.0073	0.271
PPR012887.1	CCG012530.1	0.0074 ± 0.0028	0.0196 ± 0.0075	0.3759
PPR021750.3	CCG033354.1	0.0129 ± 0.0032	0.0512 ± 0.0120	0.2517
PPR021749.1	CCG033353.1	0.0331 ± 0.0057	0.0564 ± 0.0133	0.5864
PPR018063.1	CCG029088.1	0.0143 ± 0.0041	0.0115 ± 0.0066	1.245
PPR030299.1	CCG029325.1	0.0034 ± 0.0017	0.0102 ± 0.0046	0.3354
PPR005839.1	CCG028829.1	0.0028 ± 0.0016	0.0503 ± 0.0111	0.0559

© 2015 by the authors; licensee MDPI, Basel, Switzerland. This article is an open access article distributed under the terms and conditions of the Creative Commons Attribution license (http://creativecommons.org/licenses/by/4.0/).
